# Cardiovascular metrics on CT pulmonary angiography in patients with pulmonary hypertension — re-evaluation under the updated guidelines of pulmonary hypertension

**DOI:** 10.1186/s13244-023-01535-1

**Published:** 2023-10-23

**Authors:** Anqi Liu, Wenqing Xu, Linfeng Xi, Mei Deng, Haoyu Yang, Qiang Huang, Qian Gao, Peiyao Zhang, Wanmu Xie, Zhenguo Huang, Min Liu

**Affiliations:** 1 China-Japan Friendship Hospital, Chinese Academy of Medical Sciences & Peking Union Medical College, Beijing, 100005 China; 2 Department of Radiology, Peking University China-Japan Friendship School of Clinical Medicine, Beijing, 100191 China; 3 Department of Pulmonary and Critical Care Medicine, China-Japan Friendship Hospital, Beijing, 100029 China; 4 Capital Medical University, Beijing, 100069 China; 5 Department of Radiology, China-Japan Friendship Hospital, Beijing, 100029 China

**Keywords:** Pulmonary hypertension, Computed tomography pulmonary angiography, Pulmonary artery pressure, Right heart catheterization, Hemodynamics

## Abstract

**Purpose:**

To re-assess cardiovascular metrics on computed tomography pulmonary angiography (CTPA) in predicting pulmonary hypertension (PH) under the 2022 ESC/ERS guidelines.

**Materials and methods:**

This observational study retrospectively included 272 patients (female 143, mean age = 54.9 ± 12.5 years old) with suspected PH. 218 patients were grouped to evaluate cardiovascular metrics on CTPA and develop a binary logistic regression model. The other 54 patients were grouped into the validation group to assess the performance of the prediction model under the updated criteria. Based on mean pulmonary artery pressure (mPAP), patients were divided into three groups: group A consisted of patients with mPAP ≤ 20 mmHg, group B included patients with 20 mmHg < mPAP < 25 mmHg, and group C comprised patients with mPAP ≥ 25 mmHg. Cardiovascular metrics among the three groups were compared, and receiver operating characteristic curves (ROCs) were used to evaluate the performance of cardiovascular metrics in predicting mPAP > 20 mmHg.

**Results:**

The main pulmonary arterial diameter (MPAd), MPAd/ascending aorta diameter ratio (MPAd/AAd ratio), and right ventricular free wall thickness (RVFWT) showed significant differences among the three groups (*p* < 0.05). The area under curve (AUC) of MPAd was larger than MPAd/AAd ratio and RVFWT. A MPAd cutoff value of 30.0 mm has a sensitivity of 83.1% and a specificity of 90.4%. The AUC of the binary logistic regression model (*Z* =  − 12.98187 + 0.31053 MPAd + 1.04863 RVFWT) was 0.938 ± 0.018. In the validation group, the AUC, sensitivity, specificity, and accuracy of the prediction model were 0.878, 92.7%, 76.9%, and 88.9%, respectively.

**Conclusion:**

Under the updated criteria, MPAd with a threshold value of 30.0 mm has better sensitivity and specificity in predicting PH. The binary logistic regression model may improve the diagnostic accuracy.

**Critical relevance statement:**

Under the updated criteria, the main pulmonary arterial diameter with a threshold value of 30.0 mm has better sensitivity and specificity in predicting pulmonary hypertension. The binary logistic regression model may improve diagnostic accuracy.

**Key points:**

• According to 2022 ESC/ERS guidelines, a MPAd cutoff value of 30.0 mm has better sensitivity and specificity in predicting mPAP > 20 mmHg

• A binary logistic regression model (Z = − 12.98187 + 0.31053 MPAd + 1.04863 RVFWT) was developed and had a sensitivity, specificity, and accuracy of 92.7%, 76.9%, and 88.9% in predicting mPAP > 20 mmHg.

• A binary logistic regression prediction model outperforms MPAd in predicting mPAP > 20 mmHg.

**Graphical Abstract:**

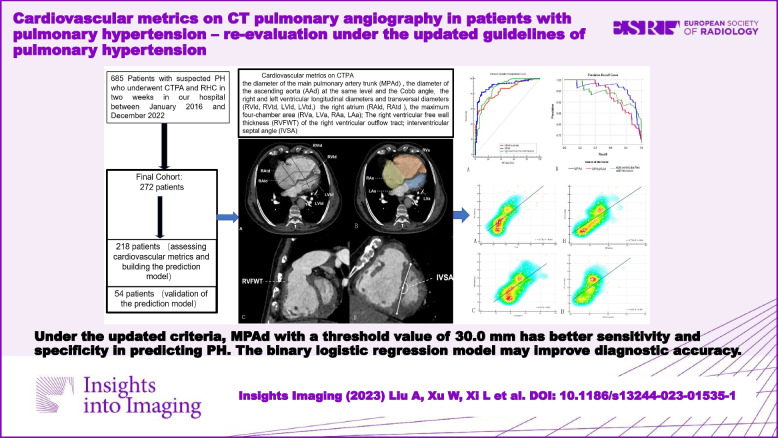

**Supplementary Information:**

The online version contains supplementary material available at 10.1186/s13244-023-01535-1.

## Introduction

Pulmonary hypertension (PH) is a hemodynamic condition with the characteristics of progressively increased pulmonary artery pressure (PAP) and pulmonary vascular resistance (PVR), finally leading to right heart failure and death [1, 2]. Different etiologies of PH have been categorized into five groups, however, the gold standard for diagnosis and evaluation of PH is right heart catheterization (RHC), which enables directly invasive assessment of pulmonary hemodynamics [1, 2]. In the past 10 years, PH is defined as an increase in mean pulmonary arterial pressure (mPAP) ≥ 25 mmHg at rest as assessed by RHC. Thus, echocardiography and computed tomography (CT) have been widely used for noninvasive assessment of PH, and cardiovascular magnetic resonance (CMR) is also increasingly being used [3–5]. Computed tomography pulmonary artery (CTPA) may raise a suspicion of PH by showing an increased main pulmonary artery diameter (MPAd) and MPAd: ascending aorta diameter ratio (MPAd/AAd ratio), enlarged right atrium and ventricle or a segmental artery: bronchus ratio 1:1 in three or four lobes [3–14].

However, available data have shown that the normal mPAP at rest is 14 ± 3 mmHg with an upper limit of normal of approximately 20 mmHg [1, 2]. In 2019, the 6th WSPH Task Force proposes to include mPAP > 20 mmHg and pulmonary vascular resistance ≥ 3 Wood Units in the definition of pre-capillary PH [3–14]. When it comes to the year of 2022, the European Society of Cardiology and the European Respiratory Society Guidelines formally updated the hemodynamics of PH, and PH is defined as an elevation of mPAP > 20 mmHg [15]. Although this change enables patients with suspected PH to receive a timely diagnosis, on the other hand, whether the old cutoff values of those cardiovascular metrics on CT in the prediction of mPAP > 20 mmHg would change has not been reported yet. Thus, we aimed to re-evaluate cardiovascular metrics on CTPA in noninvasive prediction of PH under the updated criteria of 2022 ESC/ERS guidelines to identify which metrics are capable to early detect PH and to develop and validate a new model for predicting PH based on these metrics.

## Materials and methods

### Population and study design

**Fig. 1:**
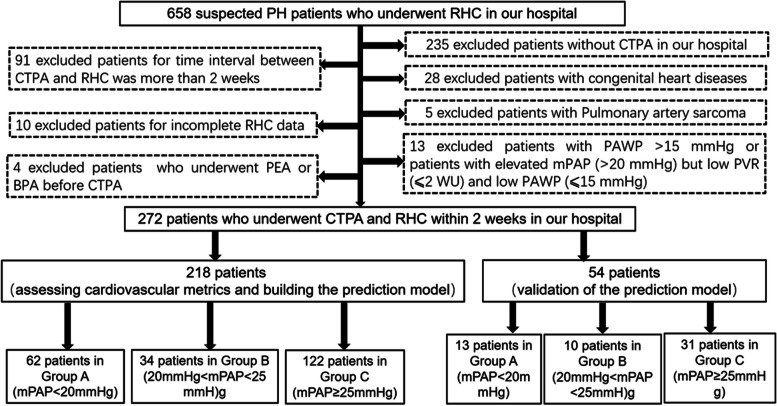
A flowchart detailing how participants were selected and grouped. PH, pulmonary hypertension; RHC, right heart catheterization; CTPA, computed tomography pulmonary angiography; PEA, pulmonary thromboendarterectomy; BPA, balloon pulmonary angioplasty; PAWP, pulmonary artery wedge pressure; mPAP, mean pulmonary artery pressure

This is a single-center retrospective study which was approved by the hospital’s Ethics Committee and was performed in accordance with the Declaration of Helsinki. Informed consent was waived for this retrospective study. Patients with suspected PH who underwent RHC in our hospital between January 2016 and December 2022 were enrolled. Patients from January 2018 to December 2022 were grouped to evaluate cardiovascular metrics on CTPA and build the prediction model. Patients from January 2016 and December 2017 were grouped into the validation group to assess the performance of the prediction model. Clinical data, CTPA, and hemodynamic metrics by RHC were collected. In the 2022 ESC/ERS Guidelines, the diagnostic criteria of pulmonary hypertension, pulmonary hypertension is defined by a mPAP > 20 mmHg at rest [15], while it was defined by a mPAP ≥ 25 mmHg before 2022 [1, 2]. The exclusion criteria were as follows: (I) patients without CTPA or RHC in our hospital; (II) the time interval between CTPA and RHC was more than 2 weeks; (III) patients with poor CTPA quality or incomplete RHC data; (IV) patients with pulmonary artery wedge pressure (PAWP) > 15 mmHg or patients with elevated mPAP (> 20 mmHg) but low PVR (≤ 2 WU) and low PAWP (≤ 15 mmHg) [15]; (V) patients with congenital heart disease; (VI) patients who underwent pulmonary thromboendarterectomy (PEA) or balloon pulmonary angioplasty (BPA) before CTPA. A total of 272 patients (female 143, mean age = 54.9 ± 12.5 years old) were finally enrolled in this study. Figure 1 demonstrates a flowchart detailing how participants were selected and grouped.

### CTPA scan protocol

All patients underwent supine CTPA with either a 256-row CT (GE Revolution CT, GE Healthcare, USA) or a 320-row CT (Aquilion ONE, Canon Medical Systems, Japan) at the end of expiration, covering the lung base to the apex. The specific scan parameters were as follows: For GE Revolution CT, tube rotation speed was 0.28 s/rotation, KV intelligent decision technology (KV assist; 100 kV and 120 kV) was used for tube voltage, 3D automatic tube current modulation (Smart-mA) was used for tube current, and the pitch was 0.992:1. Slices × collimator width were 256 × 0.625 mm, with a reconstruction image slice thickness and spacing of 0.625 mm. For Aquilion ONE, the tube rotation speed was 0.35 s/rotation, the tube voltage was 120kVp, and the automatic tube current modulation was used for tube current. Slices × collimator width were 320 × 0.5 mm, with a reconstruction image slice thickness and spacing of 0.5 mm. The contrast agent was Ultravist (370 mgI/mL, Scheringbayer, a non-ionic contrast agent, a total of 70 mL), the injection speed was 4 ~ 4.5 mL/s and 50 mL physiological saline. The contrast agent detection method used was automatically triggered, with a trigger threshold of 100 HU.

### Image reconstruction and analysis

**Fig. 2:**
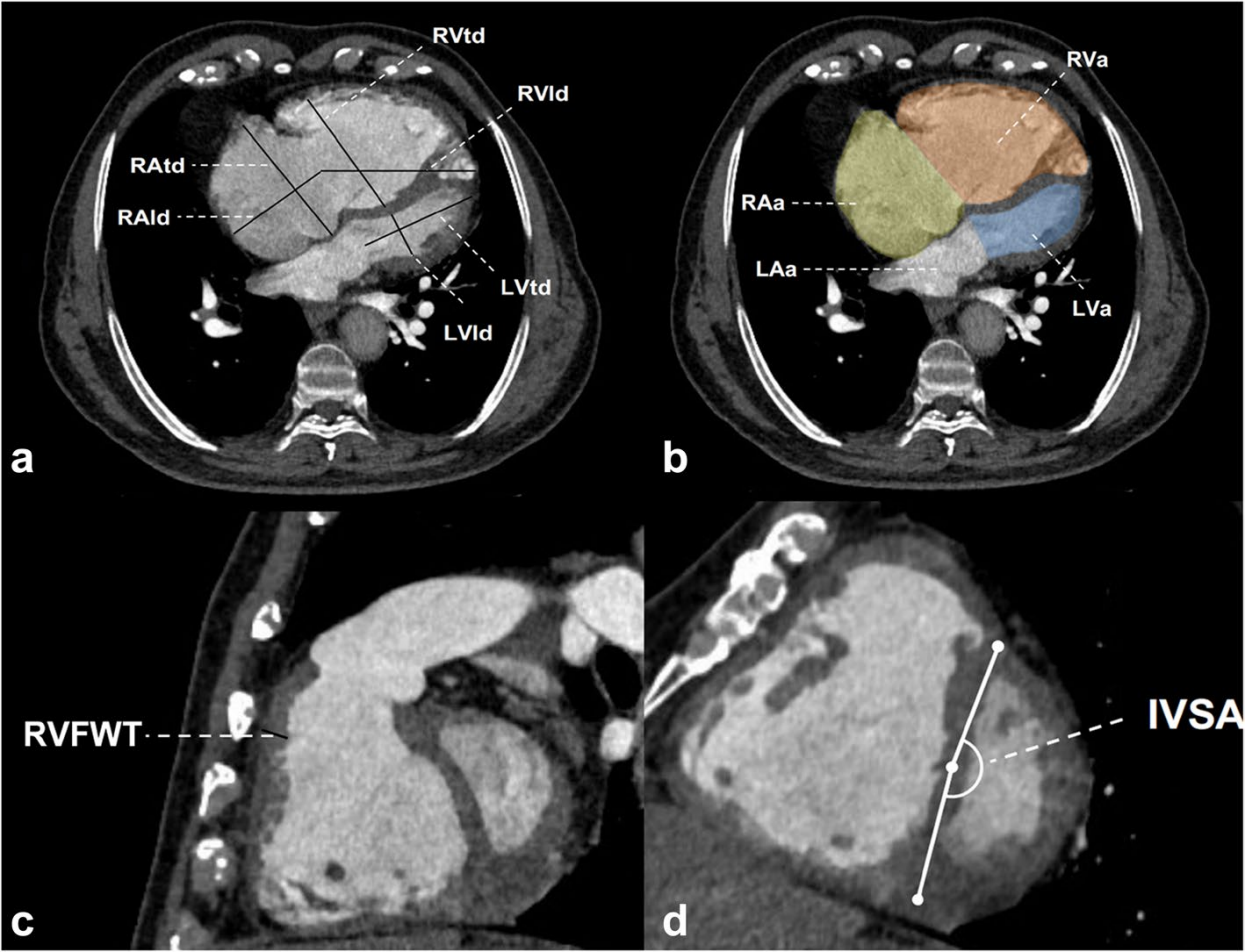
Cardiovascular metrics measured on the transversal and reconstructed views of computed tomography pulmonary angiography (CTPA). **a** The right and left ventricular longitudinal diameters and transversal diameters (RVld, RVtd, LVld, LVtd) and the right atrium (RAld, RAtd) and (**b**) the maximum four-chamber area (RVa, LVa, RAa, LAa) measured on the four-chamber cardiac view. **c** The right ventricular free wall thickness of the right ventricular outflow tract (RVFWT) and (**d**) the interventricular septal angle (IVSA) measured on the short axial cardiac view

All CTPA images were transferred to the workstation (AW4.6 GE Healthcare, USA) for the reconstruction of a four-chamber cardiac view and a short-axial two-chamber cardiac view by a radiologist with 10 years of experience. Then, cardiovascular metrics were measured by 2 radiologists with 6 years and 14 years of experience together. According to Min et al. [7, 16] MPAd and the diameter of the ascending aorta (AAd) were measured at the same level, meanwhile, the Cobb angle was the angle between the interventricular septum and the line connecting the midpoint of the sternum and the thoracic vertebral spinous process on the transversal image. Figure 2 shows the right and left ventricular longitudinal diameters and transversal diameters (RVld, RVtd, LVld, LVtd) and the right atrium (RAld, RAtd), the maximum four-chamber area (RVa, LVa, RAa, LAa) were measured on the four-chamber cardiac view [8, 9]. The right ventricular free wall thickness (RVFWT) of the right ventricular outflow tract [7] and interventricular septal angle (IVSA) were measured on the short axial two-chamber cardiac view (Fig. 2). Each cardiovascular metric is the average value of three repeated measurements.

### Right heart catheterization

All patients underwent RHC through the right internal jugular or femoral vein using a 6F Swan-Ganz catheter (Thermodilution Catheter; Bioptimal). The measured indices were right atrial pressure (RAP), pulmonary arterial pressure (PAP), pulmonary artery wedge pressure (PAWP), and pulmonary vascular resistance (PVR). Cardiac output (CO) and cardiac index (CI) were determined using the Fick method.

### Statistical analysis

The statistical analysis was performed using IBM SPSS Statistics 26.0 (SPSS Inc., New York, USA) and MedCalc (Version 20.211, MedCalc Software Ltd., Belgium). Normal data were expressed as mean ± standard deviation (SD), and one-way ANOVA and independent-sample *T* test were used for comparison in different groups; non-normal data were expressed as median with inter-quartile range (IQR:), and Kruskal–Wallis test and Mann–Whitney *U* test was used for comparison among three groups. Count data were expressed as frequency (percentage), and *χ*^2^ test was used for comparison in different groups. Receiver operator characteristic curve (ROC) and precision recall curve (PRC) were used to evaluate the performance of cardiovascular metrics in the prediction of mPAP > 20 mmHg. Binary logistic regression was used to evaluate the independent predictors for PH and to develop a prediction model. In the validation group, *Z* was calculated by applying the binary classification regression equation Z = β0 + β1 × 1 + β2 × 2 + … + β*p* × *p*. After obtaining the value of *Z*, *p* value was calculated using the logistic function *P* = 1/(1 + exp(-*Z*)). If the *p *value is greater than 0.5, the case is classified into the PH group. Sensitivity, specificity, and Youden Index of the prediction model were calculated. Overall model quality of ROC was obtained with ROC analysis on IBM SPSS Statistics 26.0. The correlations between cardiovascular metrics of CTPA and hemodynamics were analyzed using Spearman's rank correlation. Two-sided *p* < 0.05 indicated statistical significance.

## Result

### Clinical characteristics

Two hundred seventy-two patients included 75 patients in group A (mPAP ≤ 20 mmHg), 44 patients in group B (20 mmHg < mPAP < 25 mmHg), and 153 patients in group C (mPAP ≥ 25 mmHg). Patients in group A included 63 patients with chronic pulmonary embolism (CPE), 8 Takayasu arteritis, 3 fibrosing mediastinitis, and 1 Behcet syndrome. Forty-four patients with CPE were in group B. Patients in group C included 134 patients with chronic thromboembolic pulmonary hypertension, 14 idiopathic pulmonary hypertension, 4 pulmonary veno-occlusive disease, and 1 pulmonary capillary hemangiomatosis.

Among 272 patients, 218 patients (female 111, mean age = 55.4 ± 12.2 years old) were grouped in the modeling group, including 62 patients in group A, 34 patients in group B, and 122 patients in group C (mPAP ≥ 25 mmHg). The demographics, hemodynamic and clinic metrics, and clinical diagnoses for patients in three groups are shown in Table 1. Gender, body surface area (BSA), body mass index (BMI), systolic blood pressure (SBP), and diastolic blood pressure (DBP) were comparable among the three groups (*p* > 0.05). Patients in group A were younger than in groups B and C. N-terminal pro-B-type natriuretic peptide (NT-proBNP) and Six-Minute Walk Distance (6MWD) in groups A and B were comparable, and NT-proBNP was higher and 6MWD was shorter in group C compared to groups A and B. Supplement Table 1 shows the demographic characteristics and hemodynamics of PH patients in modeling group under the new and old criteria.

**Table 1:** Demographic characteristics and hemodynamics of patients in the modeling group according to mean pulmonary artery pressure

Characteristics	Group A (mPAP ≤ 20 mmHg)	Group B (25 mmHg > mPAP > 20 mmHg)	Group C (mPAP ≥ 25 mmHg)	*p* value	A and B	A and C	B and C
Case number (%)	62 (28.4%)	34 (15.6%)	122 (56%)				
Age (years)	52.1 ± 12.6	57.9 ± 14.0	56.3 ± 11.3	0.035	0.026	0.025	0.513
Gender (male/female)	29/33	18/16	56/66	0.57	0.979	0.404	0.467
BMI (kg/m^2^)	24.9 ± 3.5	25.5 ± 4.5	24.3 ± 3.2	0.227	0.585	0.053	0.141
BSA (m^2^)	1.76 ± 0.19	1.76 ± 0.18	1.70 ± 0.18	0.075	0.975	0.043	0.149
SBP (mmHg)	133.2 ± 20.3	129.9 ± 18.3	130.5 ± 19.8	0.717	0.549	0.448	0.902
DBP (mmHg)	81.3 ± 12.7	80.3 ± 11.5	83.9 ± 13.9	0.386	0.799	0.269	0.296
NT-proBNP (pg/mL)	54 (20–83)	70 (38.8–121.5)	519 (179–1284)	< 0.001*	0.377	< 0.001*	< 0.001*
Heart rate (bpm)	70 ± 11	66 ± 7	74 ± 13	0.03	0.239	0.106	0.016
6MWD (m)	530 (442–580)	472.0 (430–533.5)	393.5 (300–460)	< 0.001*	0.614	< 0.001*	< 0.001*
**Hemodynamics**							
MPA SO%	74.2 ± 6.4	71.6 ± 3.8	65.8 ± 9.8	< 0.001*	0.412	0.009	0.004
Aorta SO%	99.4 ± 1.3	98.9 ± 2.0	98.3 ± 2.8	0.03	0.412	0.009	0.317
mPAP (mmHg)	15.2 ± 3.2	22.9 ± 1.1	40.6 ± 10.6	< 0.001*	< 0.001*	< 0.001*	< 0.001*
mRAP (mmHg)	2 (0.8–4)	3 (1–5)	5 (2–7)	< 0.001*	0.032	< 0.001*	0.062
mRVP (mmHg)	9.5 (8–12)	14 (12–17)	26 (21–31)	< 0.001*	0.017	< 0.001*	< 0.001*
PAWP (mmHg)	9.2 ± 3	11.1 ± 2.3	9.4 ± 2.8	0.017	0.008	0.76	0.007
PVR (Wood U)	1.3 (0.7–1.8)	2.6 (2.0–2.8)	9.6 (6.3–13.8)	< 0.001*	0.033	< 0.001*	< 0.001*
CO (L/min)	4.6 ± 1.3	3.9 ± 0.7	3.3 ± 1.0	< 0.001*	0.012	< 0.001*	0.025
CI (L/min/m^2^)	2.6 ± 0.7	2 .2 ± 0.4	2.0 ± 0.6	< 0.001*	0.009	< 0.001*	0.04
**Diagnosis**							
CPE	55	34	113				
Takayasu arteritis	4						
Fibrosing mediastinitis	2						
Behcet syndrome	1						
IPAH			5				
PVOD			3				
PCH			1				

*CTEPH* Chronic thromboembolic pulmonary hypertension, *BMI* Body mass index, *NT-proBNP* N-terminal-pro-B-type natriuretic peptide, *6MWD* 6-min walking distance, *NYHA FC* New York Heart Association classification functional class, *mPAP* mean pulmonary arterial pressure, *mRAP* mean right atrial pressure, *PAWP* Pulmonary artery wedge pressure, *SBP* Systolic blood pressure, *DBP* Diastolic blood pressure, *PVR* Pulmonary vascular resistance, *CO* Cardiac output, *CI* Cardiac index, *CPE* Chronic pulmonary embolism, *IPAH* Idiopathic pulmonary hypertension, *PVOD* Pulmonary veno-occlusive disease, *PCH* Pulmonary capillary hemangiomatosis

*p < 0.001

**Fig. 3:**
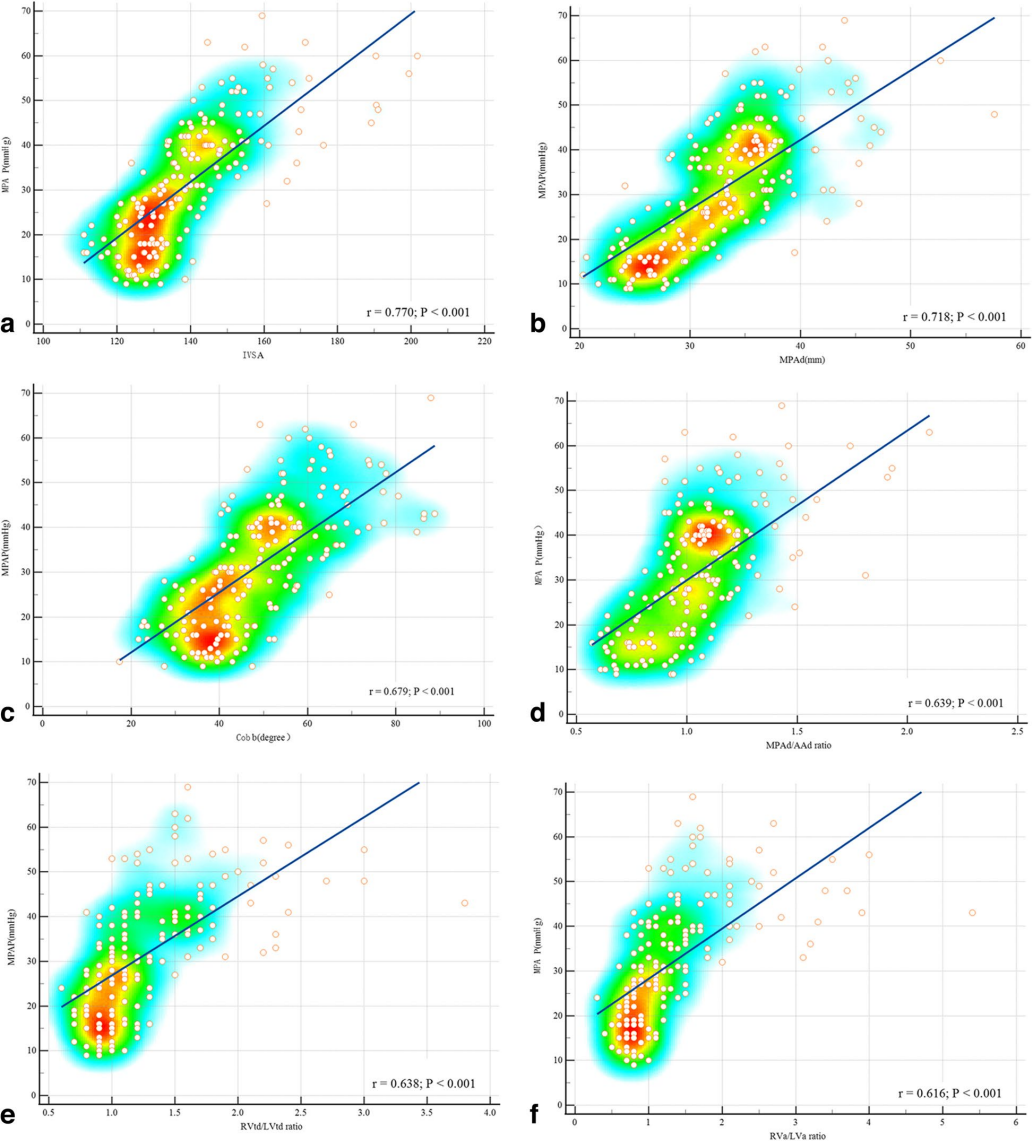
Scatter diagram and heatmap of cardiovascular parameters and mean pulmonary artery pressure(mPAP). **a** Interventricular septal angle (IVSA). **b** Main pulmonary artery diameter (MPAd). **c** Cobb angle. **d** MPAd/AAd ratio. **e** RVtd/LVtd ratio. **f** RVa/LVa ratio positively correlates with mPAP

**Fig. 4:**
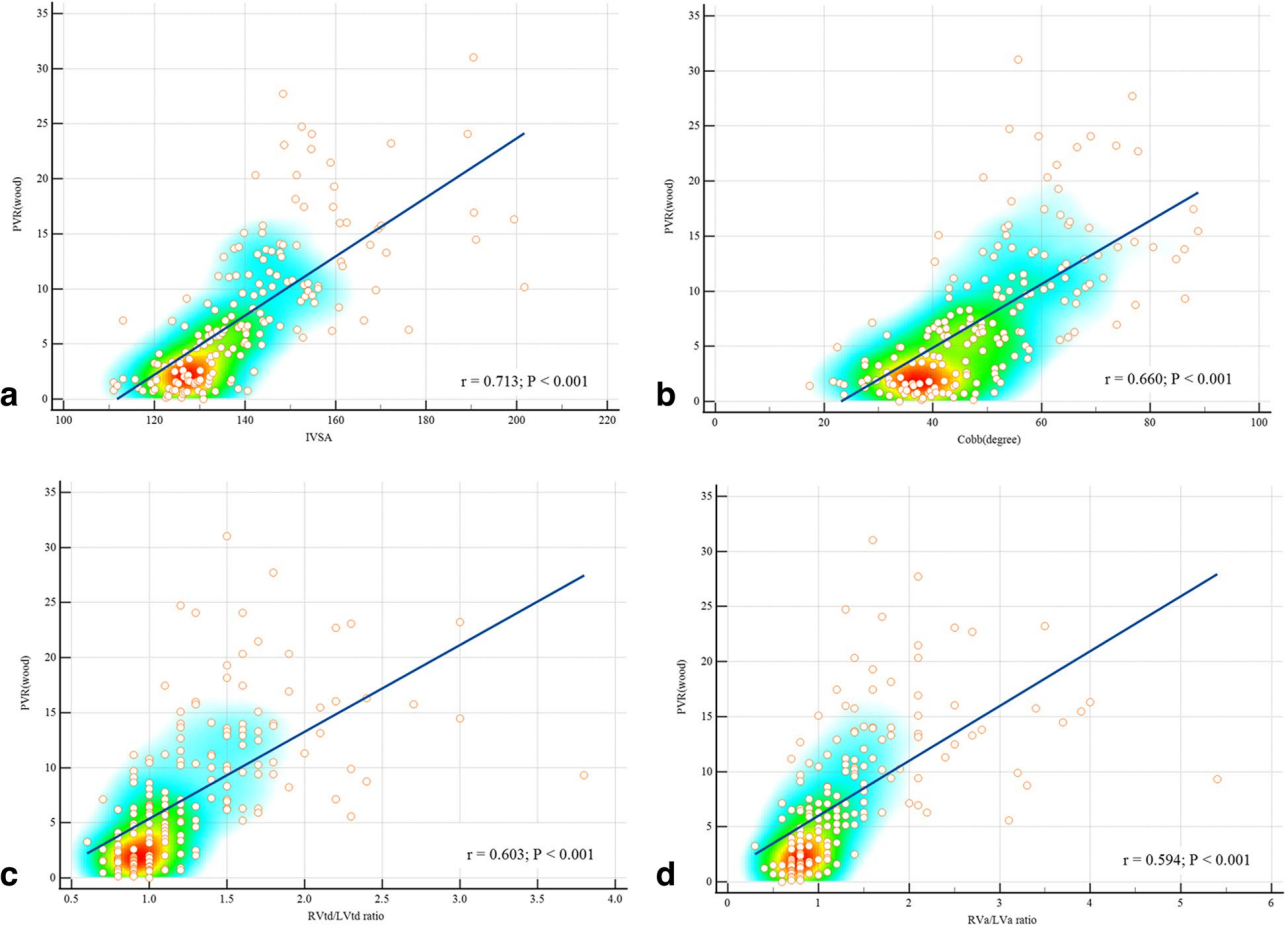
Scatter diagram and heatmap of cardiovascular parameters and pulmonary vascular resistance (PVR). **a** Interventricular septal angle (IVSA). **b** Cobb angle. **c** RVtd/LVtd ratio. **d** RVa/LVa ratio positively correlates with PVR

Figure 3 indicates that IVSA (*r* = 0.770, *p* < 0.001), MPAd (r = 0.718, *p* < 0.001), Cobb angle (*r* = 0.679, *p* < 0.001), MPAd/AAd ratio (*r* = 0.639, *p* < 0.001), RVtd/LVtd ratio (*r* = 0.638, *p* < 0.001), RVa/LVa ratio (*r* = 0.616, *p* < 0.001), and RVFWT (*r* = 0.556, *p* < 0.001) all correlate with mPAP. Figure 4 depicts that IVSA (*r* = 0.713, *p* < 0.001), Cobb angle (*r* = 0.660, *p* < 0.001), RVtd/LVtd ratio (*r* = 0.603, *p* < 0.001), RVa/LVa ratio (*r* = 0.594, *p* < 0.001), MPAd (*r* = 0.513, *p* < 0.001), MPAd/AAd ratio (*r* = 0.426, *p* < 0.001), and RVFWT (*r* = 0.395, *p* < 0.001) are all positively correlated with PVR.

### Comparison of cardiovascular metrics on CTPA among three groups

Cardiovascular metrics among the three groups are shown in Table 2. MPAd, MPAd/AAd ratio, and RVFWT significantly differed among the three groups. AAd and LAa were comparable among the three groups. There were significant differences among MPAd, MPAd/AAd ratio, and RVFWT in the three groups, while AAd and LAa were found to be similar among the three groups. The other cardiovascular metrics, such as RVtd, RVld, RAtd, RAld, RVa, RAa, septal angle, IVSA, RVtd/LVtd ratio, and RVa/LVa ratio in group A and group B were similar and lower as compared to those in group C. LVtd and LVa in both groups A and B were comparable but higher than those in group C.

**Table 2:** Comparison of cardiovascular metrics on CTPA in patients among three groups

Cardiovascular metrics on CTPA	Group A (mPAP ≤ 20 mmHg)	Group B (25 mmHg > mPAP > 20 mmHg)	Group C (mPAP ≥ 25 mmHg)	*p* value	A and B	A and C	B and C
*MPAd (mm)*	*26.4* ± *3.6*	*30.5* ± *5.0*	*36.1* ± *5.2*	< 0.001*	< *0.001*	< *0.001*	*0.001*
AAd (mm)	32.1 ± 5.2	33.2 ± 6.0	32.1 ± 4.7	0.487	0.325	0.902	0.328
RVtd (mm)	36.4 ± 4.5	37.6 ± 7.2	49.0 ± 9.5	< 0.001	0.493	< 0.001	< 0.001
RVld (mm)	67.8 ± 9.9	68.9 ± 11.4	75.8 ± 9.1	< 0.001	0.571	< 0.001	< 0.001
LVtd (mm)	40.8 ± 7.4	40.1 ± 8.7	35.8 ± 7.9	0.004	0.923	< 0.001	0.001
LVld (mm)	71.2 ± 9.0	70.2 ± 8.6	67.1 ± 9.7	0.013	0.571	0.005	0.101
RVa (mm^2^)	19.1 ± 5.1	20.0 ± 7.1	30.3 ± 9.0	< 0.001	0.612	< 0.001	< 0.001
RAa (mm^2^)	15.3 ± 5.9	18.2 ± 6.6	25.3 ± 10.9	< 0.001	0.138	< 0.001	0.001
LVa (mm^2^)	25.2 ± 6.6	24.6 ± 7.2	21.3 ± 6.8	< 0.001	0.676	< 0.001	0.013
LAa (mm^2^)	17.5 ± 4.9	17.7 ± 6.1	16.5 ± 5.2	0.582	0.609	0.586	0.317
RAtd(mm)	43.0 ± 6.3	46.8 ± 8.6	56.2 ± 11.9	< 0.001	0.082	< 0.001	< 0.001
*RVFWT (mm)*	*3.3* ± *1.1*	*4.9* ± *1.0*	*5.6* ± *1.6*	< 0.001*	< *0.001*	< *0.001*	*0.023*
*MPAd/AAd ratio*	*0.84* ± *0.14*	*0.94* ± *0.21*	*1.16* ± *0.25*	< 0.001*	*0.027*	< *0.001*	< *0.001*
RVtd/LVtd ratio	0.91 ± 0.13	0.94 ± 0.18	1.46 ± 0.50	< 0.001	0.73	< 0.001	< 0.001
RAtd/LAtd ratio	1.22 ± 0.20	1.28 ± 0.27	1.16 ± 0.20	0.016	0.247	0.069	0.01
RVa/LVa ratio	0.78 ± 0.16	0.83 ± 0.29	1.57 ± 0.81	< 0.001	0.782	< 0.001	< 0.001
Cobb angle (degree)	38.0 ± 8.1	42.6 ± 10.8	55.8 ± 12.9	< 0.001	0.059	< 0.001	< 0.001
IVSA (degree)	125.9 ± 6.9	128.3 ± 7.7	147.6 ± 16.6	< 0.001	0.375	< 0.001	< 0.001

*MPAd* diameter of main pulmonary artery, *AAd* Ascending aorta diameter, *RVtd* Right ventricular transversal diameter, *RVld* Right ventricular longitudinal diameter, *RAtd* Right atrial transversal diameter, *LAtd* Left atrial transversal diameter, *LVtd* Left ventricular transversal diameter, *LVld* Left ventricular longitudinal diameter, *RVa* Right ventricular area, *RAa* Right atrial area, *LVa* Left ventricular area, *LAa* Left atrial area, *RVFWT* Right ventricular free wall thickness, *IVSA* Interventricular septal angle; *p < 0.001 among three groups

### Cardiovascular metrics and prediction model in prediction of PH

**Fig. 5:**
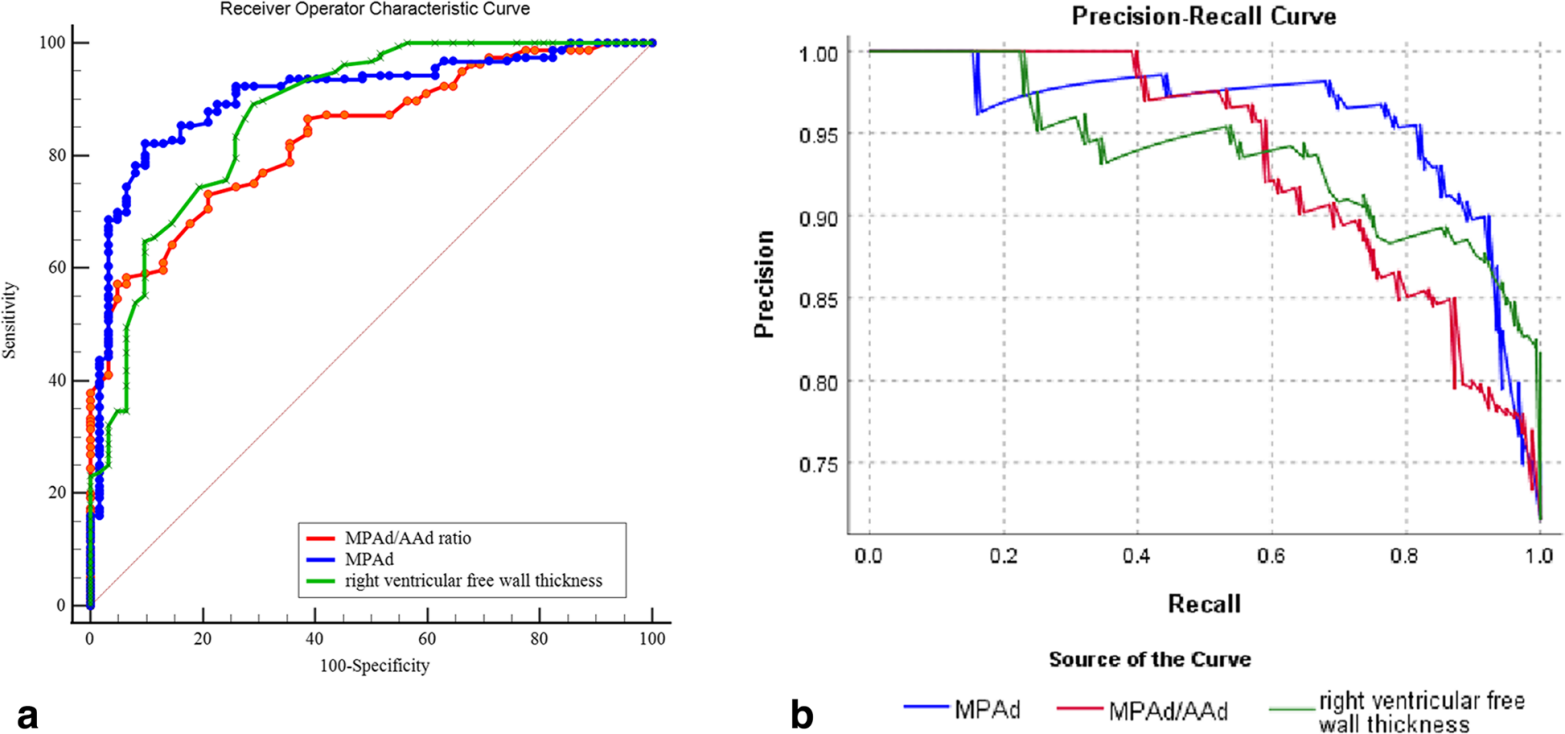
Performance of MPAd, MPAd/AAd ratio, and RVFWT in prediction of PH in mean pulmonary artery pressure (mPAP) > 20 mmHg. **a** Area under receiver operating characteristic curves (ROCs) of MPAd is greater than RVFWT (*z* = 2.813, *p* = 0.005) while AUCs between RVFWT and MPAd/AAd ratio are comparable. **b** Precision recall curves (PRCs) of MPA, RVFWT, and MPAd/AAd ratio and PRC of MPAd is located in the upper right corner with a higher PRAUC compared to that of MPAd/AAd and RVFWT

**Fig. 6:**
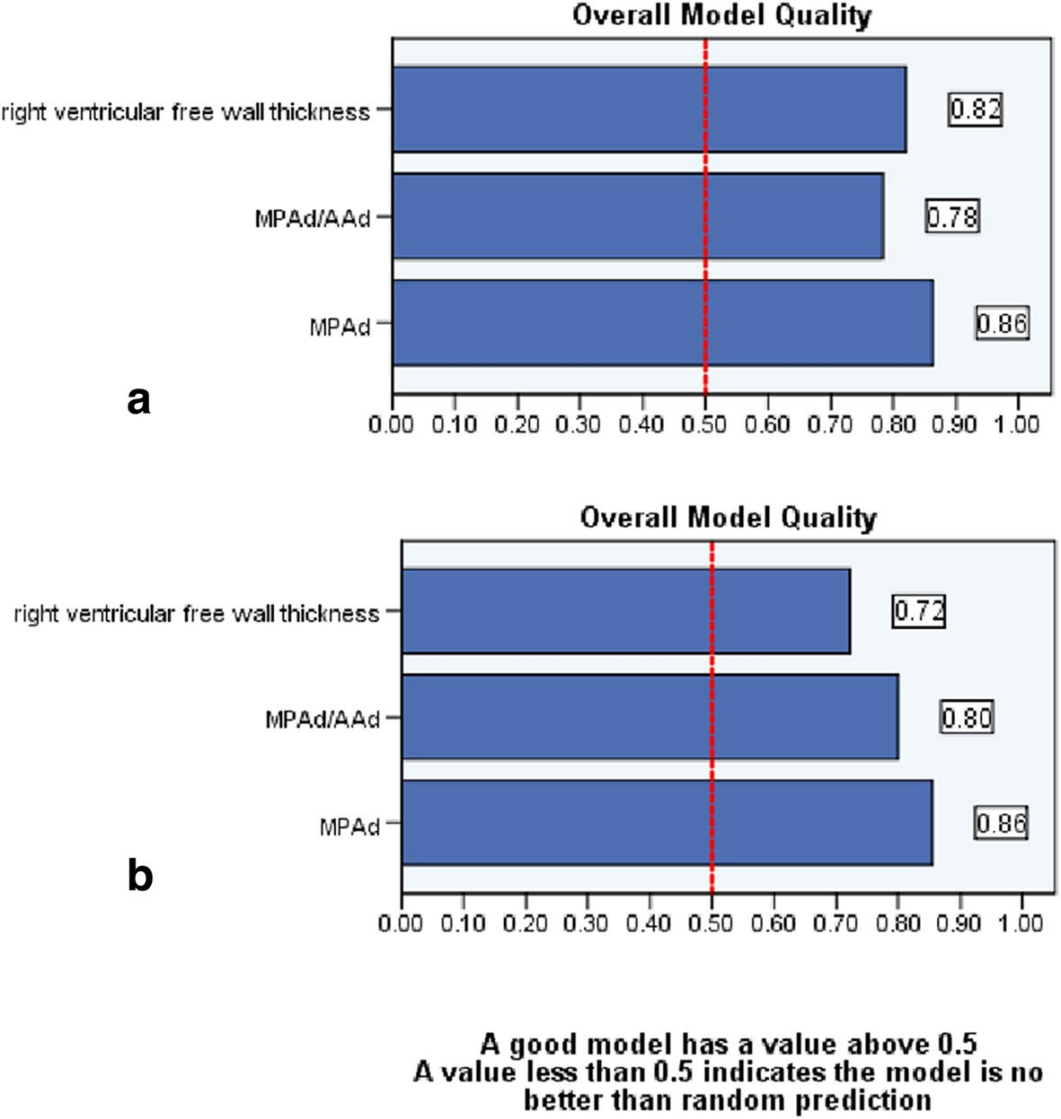
The overall model quality of MPAd, MPAd/Aad ratio, and RVFWT in the prediction of pulmonary hypertension. **a** MPAd outperforms RVFWT and MPAd/AAd ratio under the updated criteria. **b** MPAd outperforms MPAd/AAd ratio and RVFWT under the old criteria

Table 3 reveals that MPAd has a higher AUC than other metrics for predicting PH, regardless of whether it is the updated or old criteria. Additionally, using the updated criteria, an MPA cutoff value of 30.0 mm has a sensitivity of 83.1% and a specificity of 90.4%. Figure 5 shows that the AUC of MPAd is greater than RVFWT (*z* = 2.813, *p* = 0.005) while AUCs between RVFWT and MPAd/AAd ratio are comparable (*z* = 1.068, *p* = 0.285). Figure 5 demonstrates that PRCs of MPAd, RVFWT, and MPAd/AAd ratio with their AUPRC respectively are 0.958 (95% CI 0.913, 0.980), 0.937 (95% CI 0.886, 0.996), and 0.933 (95% CI 0.882, 0.963). PRC of MPAd was located in the upper right corner with a higher PRAUC compared to that of MPAd/AAd and RVFWT. According to the overall model quality in Fig. 6, MPAd outperforms RVFWT and MPAd/AAd ratio, regardless of whether the updated or old criteria were used. Binary logistic regression analysis indicated that both MPAd (OR = 1.317, 95% CI 1.174–1.478, *p* < 0.01) and RVFWT (OR = 2.817, 95% CI 1.760–4.501, *p* < 0.001) were independent predictors of CTPA for predicting PH under the new criteria. The binary logistic regression prediction model was *Z* =  − 12.98187 + 0.31053 MPAd + 1.04863 RVFWT, and its AUC was 0.938 ± 0.018 (95% CI 0.897–0.996).

**Table 3:** ROCs of cardiovascular metrics and prediction model in diagnosis of PH under the updated and old diagnostic criteria

Cardiovascular metrics	Cut-off value	AUC of ROC	95% CI		*p* value	Sensitivity %	Specificity %	Youden Index
			Lower limit	Upper limit				
CTPA metrics for mPAP > 20 mmHg								
MPAd (mm)	30.0	0.906 ± 0.022	0.860	0.942	< .001*	83.1	90.4	0.735
RVFWT (mm)	3.8	0.874 ± 0.028	0.822	0.915	< .001*	89.1	71.0	0.601
MPAd/AAd ratio	1.1	0.836 ± 0.027	0.780	0.883	< .001*	57.1	95.2	0.522
Predicting model▲	-	0.938 ± 0.018	0.897	0.996	< .001*	-	-	
CTPA metrics for mPAP ≥ 25 mmHg								
MPAd (mm)	30.4	0.899 ± 0.022	0.851	0.936	< .001*	91.8	77.1	0.689
RVWT (mm)	4.8	0.783 ± 0.031	0.722	0.836	< .001*	64.8	81.3	0.460
MPAd/AAd ratio	1.0	0.850 ± 0.026	0.796	0.895	< .001*	77.1	76.0	0.531
Predicting model▼	-	0.919 ± 0.021	0.875	0.952	< .001*	-	-	

*PH* Pulmonary hypertension, *ROC* Receiver operating characteristic curve, *AUC* Area under the curve, *CTPA* Computed tomography pulmonary angiography, *MPAd* the main pulmonary arterial diameter, *MPAd/AAd ratio* MPAd/ascending aorta diameter ratio, *RVFWT* right ventricular free wall thickness; predicting model binary logistic regression

▲ MPAd and RVWT; ▼ MPAd and MPAd/AAd ratio and RVWT; *p < 0.001

### Validation of the prediction model

Fifty-four patients (female 32, mean age = 53.4 ± 13.3 years old) entered in the validation group, including 13 patients (8 patients with CPE, 4 Takayasu arteritis, and 1 fibrosing mediastinitis) in group A, 10 patients with CPE in group B, and 31 patients (21patients with CTEPH, 9 with IPAH and 1 PVOD) in group C. The binary logistic regression model identified 38 true positive cases, 3 false negative cases, 10 true negative cases, and 3 false positive cases. The sensitivity, specificity, accuracy, and Youden’s Index of the prediction model were 92.7%, 76.9%, 88.9%, and 0.696, respectively. AUC and the overall model quality of the binary logistic regression model were 0.878 ± 0.062 and 0.76, which were higher than those of MPAd (0.841 ± 0.067 and 0.71) (Supplement Fig. 1).

## Discussion

In this study, we re-evaluated cardiovascular metrics on CTPA in the prediction of PH using the updated diagnostic criteria of the 2022 ESC/ERS Guidelines [15]. And there are several major findings: (I) both MPAd and RVFWT were identified as independent predictors of CTPA to predict PH under the updated criteria; (II) the cutoff value of 30.0 mm for MPAd demonstrated high sensitivity and specificity in predicting PH under the updated criteria; (III) we develop and validate a new binary logistic regression prediction model (*Y* =  − 12.98187 + 0.31053 MPAd + 1.04863 RVFWT) to predict PH under the updated criteria.

Although RHC has been the gold standard for the diagnosis of PH, non-invasive imaging metrics obtained from various modalities, such as echocardiography, CT, and MRI, have been widely utilized in clinical practice for evaluating PH [16]. Previous studies have demonstrated that some metrics such as MPAd, MPAd/AAd ratio, and septal angle can help detect PH. The diagnostic cutoff values for cardiovascular metrics on CTPA varied with the diagnostic criteria used. Under the old diagnostic criteria for PH ( mPAP ≥ 25 mmHg), a cut-off value of 29 mm for MPAd has been used as an indicator of PH [17]. In addition, MPAd/AAd ratio > 1 has also been shown to be highly indicative of PH [18]. Liu et al. reported that a septal angle > 68° could be used as a predictor of PVR > 1000 dyn s cm^−5^ [19]. In 2022, the European Society of Cardiology and the European Respiratory Society Guidelines (2022 ESC/ERS Guidelines) proposed a formal update for the hemodynamics of PH. The updated definition for PH is mPAP > 20 mmHg [15]. Since mPAP decreased from 25 to 20 mmHg, it is necessary to re-assess the impact of the change in diagnostic criteria of mPAP to cardiovascular metrics on CTPA, we compared cardiovascular metrics among the three groups: group A (mPAP ≤ 20 mmHg), group B (20 mmHg < mPAP < 25 mmHg), and group C (mPAP ≥ 25 mmHg). We found that MPAd, MPAd/AAd ratio, and RVFWT increased as mPAP levels increased, with significant statistical differences observed among the three groups. However, other cardiovascular metrics were comparable between group A and group B. This indicated that MPAd, MPAd/AAd ratio, and RVFWT could be used to predict mPAP > 20 mmHg. The cut-off value for MPAd in PH patients is 30.0 mm for the updated criteria, with a high sensitivity of 83.1% and specificity of 90.4%. Similarly, Swift et al. [6] proposed that a pulmonary artery diameter of 30 mm represents a compromise threshold for identifying patients with mPAP > 20 mmHg. Moreover, according to the old criteria, we found that the cut-off value for MPAd in PH patients was 30.4 mm, with a high sensitivity of 91.8% and moderate specificity of 77.1%. The above results could potentially explain the inconsistent results obtained from using static pulmonary artery (PA) dimensions on routine chest CT scans for the diagnosis of PH [16].

A recent meta-analysis [11] including ten studies from different PH groups (mPAP ≥ 25 mmHg) showed that the pooled sensitivity, specificity, and AUC of MPAd/AAd ratio for identifying PH were 65.2%, 83%, and 0.84, respectively, with a cut-off value of ≥ 1. Similar to our research, a cut-off value of MPAd/AAd ratio ≥ 1 showed sensitivity, specificity, and AUC of 77.1%, 76%, and 0.850 ± 0.026, respectively. When using the updated criteria, the MPAd/AAd ratio revealed a cut-off value of ≥ 1.1, which was associated with a decreased sensitivity and an elevated specificity, and the AUC remained comparable to the performance of the MPAd/AAd ratio under the old criteria. The RVFWT cut-off value of ≥ 3.8 mm has shown a good sensitivity in predicting mPAP > 20 mmHg, but the specificity was somewhat insufficient. On the other hand, the RVFWT cut-off value of ≥ 4.8 mm has poor sensitivity and moderate specificity in the prediction of mPAP > 25 mmHg.

Due to the limited number of cases with mPAP ≤ 20 mmHg, PRC is more informative than the ROC when evaluating classifiers on imbalanced datasets [20, 21]. Therefore, we further compare PRCs of MPAd, MPAd/AAd ratio, and RVFWT in identifying PH under the updated criteria. Our results indicated that the PRC of MPAd was located in the upper right corner with a higher PRAUC compared to that of MPAd/AAd and RVFWT. Furthermore, whether the updated or the old criteria were used, MPAd was the best indicator for evaluating PH. Although MPAd, MPAd/AAd ratio, and RVFWT showed significant differences among the three groups, and MPAd had the highest AUC and PRAUC, binary logistic regression analysis showed that under the updated criteria, MPAd and RVFWT were independent predictors of pulmonary hypertension, while under the old criteria, MPAd, MPAd/AAd ratio, and RVFWT were independent predictors. Importantly, in comparison to the performance of each independent predictor, the binary logistic regression prediction model (*Y* =  − 12.98187 + 0.31053 MPA + 1.04863 RVFWT) demonstrated a further improvement in AUC, indicating that using a combination of variables was superior to each independent predictor alone and may improve diagnostic accuracy. Furthermore, this study demonstrated that apart from MPAd, Cobb angle derived from transversal views, RVtd/LVtd and RVa/LVa measured from the four-chamber view, as well as IVSA and RVFWT measured from the short-axis view, were all significantly correlated with two most important pulmonary artery hemodynamics including mPAP and PVR.

## Limitations

There are several limitations in our research. First, this was a single-center retrospective study in a large tertiary hospital which only included patients with pre-capillary PH, and most of them were CTEPH and CPE, which inevitably limits the generalizability of these findings to other types of PH. The second major limitation is the small number of patients with normal pulmonary pressure (mPAP ≤ 20 mmHg) and those with mPAP between 20 and 25 mmHg. This is because RHC is not a routine procedure for healthy individuals. Therefore, the findings of this study may be underestimated. To improve the study’s external validity, future research should include a larger sample size of patients with varying degrees of mPAP. Finally, it should be noted that the CTPA was not scanned using an ECG-gating protocol. As a result, certain cardiovascular metrics, including right atrial and ventricular diameter and area, as well as right ventricular wall thickness, were not measured in the same phase, such as the systolic phase. This may have affected the accuracy and reliability of these measurements. CTPA with an ECG-gating protocol may improve the accuracy of cardiovascular metric measurement in the prediction of PH.

## Conclusion

Whether using the old or updated criteria, MPAd is superior to MRAd/AAd ratio and RVFWT in predicting PH. MPA's cutoff value of 30.0 mm has better specificity and sensitivity under the updated criteria. Notably, the performance of the binary logistic regression prediction model may improve diagnostic accuracy.

### Supplementary Information

**Additional file 1:** **Supplementary Table 1.** The clinical characteristics of patients with PH under the new and old criteria. **Supplementary Figure 1.** AUC and over all model quality of ROC of the binary logistic regression model and main pulmonary arterial diameter (MPAd) in diagnosis of pulmonary hypertension under the updated criteria. (A). Area of ROC of binary logistic regression model and MPAd respectively is 0.878±0.062 and 0.841±0.067. (B) over all model quality of the binary logistic regression model and main pulmonary arterial diameter (MPAd) respectively is 0.76 and 0.71.

## Data Availability

The datasets generated during and/or analyzed during the current study are available from the corresponding author upon reasonable request.

## Abbreviations

6MWD: Six-Minute Walk Distance
AAd: Ascending aorta diameter
BMI: Body mass index
BPA: Balloon pulmonary angioplasty
BSA: Body surface area
CI: Cardiac Index
CO: Cardiac output
CPE: Chronic pulmonary embolism
CTEPH: Chronic thromboembolic pulmonary hypertension
CTPA: Computed tomography pulmonary angiography
DBP: Diastolic blood pressure
IPAH: Idiopathic pulmonary hypertension
IVSA: Interventricular septal angle
LAa: The left atrium area
LVa: The left ventricular area
LVld: The left ventricular longitudinal diameters
LVtd: The left ventricular transversal diameters
MPAd: Main pulmonary arterial diameter
mPAP: Mean pulmonary artery pressure
NT-proBNP: N-terminal pro-B-type natriuretic peptide
PAP: Pulmonary artery pressure
PAWP: Pulmonary artery wedge pressure
PCH: Pulmonary capillary hemangiomatosis
PEA: Pulmonary thromboendarterectomy
PH: Pulmonary hypertension
PRC: Precision recall curve
PVOD: Pulmonary veno-occlusive disease
PVR: Pulmonary vascular resistance
RAa: The right atrium area
Rald: The right atrium longitudinal diameters
RAtd: The left atrium transversal diameters
RHC: Right heart catheterization
ROC: Receiver operating characteristic curve
RVa: The right ventricular area
RVFWT: Right ventricular free wall thickness
RVld: The right ventricular longitudinal diameters
RVtd: The right ventricular transversal diameters
SBP: Systolic blood pressure
