# Supplementary material for: Cardiovascular metrics on CT pulmonary angiography in patients with pulmonary hypertension — re-evaluation under the updated guidelines of pulmonary hypertension
Source: Insights Imaging. 2023 Oct 23;14:179. doi: 10.1186/s13244-023-01535-1 (PMC10593727; doi:10.1186/s13244-023-01535-1)
Supplement: Supplementary file 1 — Additional file 1: Supplementary Table 1. The clinical characteristics of patients with PH under the new and old criteria. Supplementary Figure 1. AUC and over all model quality of ROC of the binary logistic regression model and main pulmonary arterial diameter (MPAd) in diagnosis of pulmonary hypertension under the updated criteria. (A). Area of ROC of binary logistic regression model and MPAd respectively is 0.878±0.062 and 0.841±0.067. (B) over all model quality of the binary logistic regression model and main pulmonary arterial diameter (MPAd) respectively is 0.76 and 0.71. [file 13244_2023_1535_MOESM1_ESM.docx]

**Cardiovascular metrics on CT pulmonary angiography in patients with pulmonary hypertension - Re-evaluation under the updated guidelines of pulmonary hypertension**

**ELECTRONIC SUPPLEMENTARY MATERIAL**

**Supplementary Table 1. The clinical characteristics of patients with PH under the new and old criteria.**

| Characteristics | New criteria  mPAP>20mmHg | Old criteria  mPAP≥25mmHg |
| --- | --- | --- |
| Case number | 156 | 122 |
| Age (years) | 56.7±11.9 | 56.3 ±11.3 |
| Gender (Male/Female) | 74/82 | 56/66 |
| BMI (Kg/m^2^) | 24.5±3.5 | 24.3 ± 3.2 |
| BSA(m^2^) | 1.7±0.21 | 1.70±0.18 |
| SBP (mmHg) | 130.4±19.5 | 130.5 ± 19.8 |
| DBP (mmHg) | 83.4±13.6 | 83.9 ± 13.9 |
| NT-proBNP (pg/ml) | 399(105-1196) | 519(179-1284) |
| Heart Rate (bpm) | 73±12 | 74±13 |
| 6MWD (m) | 430(321-473) | 393.5(300-460) |
| **Hemodynamics** |  |  |
| MPA SO% | 66.7±9.3 | 65.8±9.8 |
| aorta SO% | 98.4±2.7 | 98.3±2.8 |
| mPAP (mmHg) | 36.7±11.9 | 40.6 ±10.6 |
| mRAP (mmHg) | 5(2-7) | 5 (2-7) |
| mRVP (mmHg) | 25(18-30） | 26(21-31) |
| PAWP (mmHg) | 9.7±2.6 | 9.4 ±2.8 |
| PVR (Wood U) | 8.1(5.1-13.2） | 9.6(6.3-13.8) |
| CO (L/min) | 3.3±1.1 | 3.3 ±1.0 |
| CI (L/min/m2) | 2.0±0.5 | 2.0 ± 0.6 |
| **Diagnosis** |  |  |
| CPE | 89 | 113 |
| Takayasu arteritis | 4 | 0 |
| fibrosing mediastinitis | 2 | 0 |
| Behcet syndrome | 1 | 0 |
| IPAH | 0 | 5 |
| PVOD | 0 | 3 |
| PCH | 0 | 1 |

Note. CTEPH=chronic thromboembolic pulmonary hypertension; BMI=body mass index; NT-proBNP=N-terminal-pro-B-type natriuretic peptide; 6MWD=6-minute walking distance; NYHA=FC New York Heart Association classification functional class; mPAP=mean pulmonary arterial pressure; mRAP=mean right atrial pressure, PAWP=pulmonary artery wedge pressure; SBP=systolic blood pressure; DBP=diastolic blood pressure; PVR=pulmonary vascular resistance; CO=cardiac output; CI=cardiac index; CPE=chronic pulmonary embolism; IPAH=idiopathic pulmonary hypertension; PVOD= pulmonary veno-occlusive disease; PCH=pulmonary capillary haemangiomatosis

**Supplementary Figure 1.** AUC and over all model quality of ROC of the binary logistic regression model and main pulmonary arterial diameter (MPAd) in diagnosis of pulmonary hypertension under the updated criteria. (A). Area of ROC of binary logistic regression model and MPAd respectively is 0.878±0.062 and 0.841±0.067. (B) over all model quality of the binary logistic regression model and main pulmonary arterial diameter (MPAd) respectively is 0.76 and 0.71.


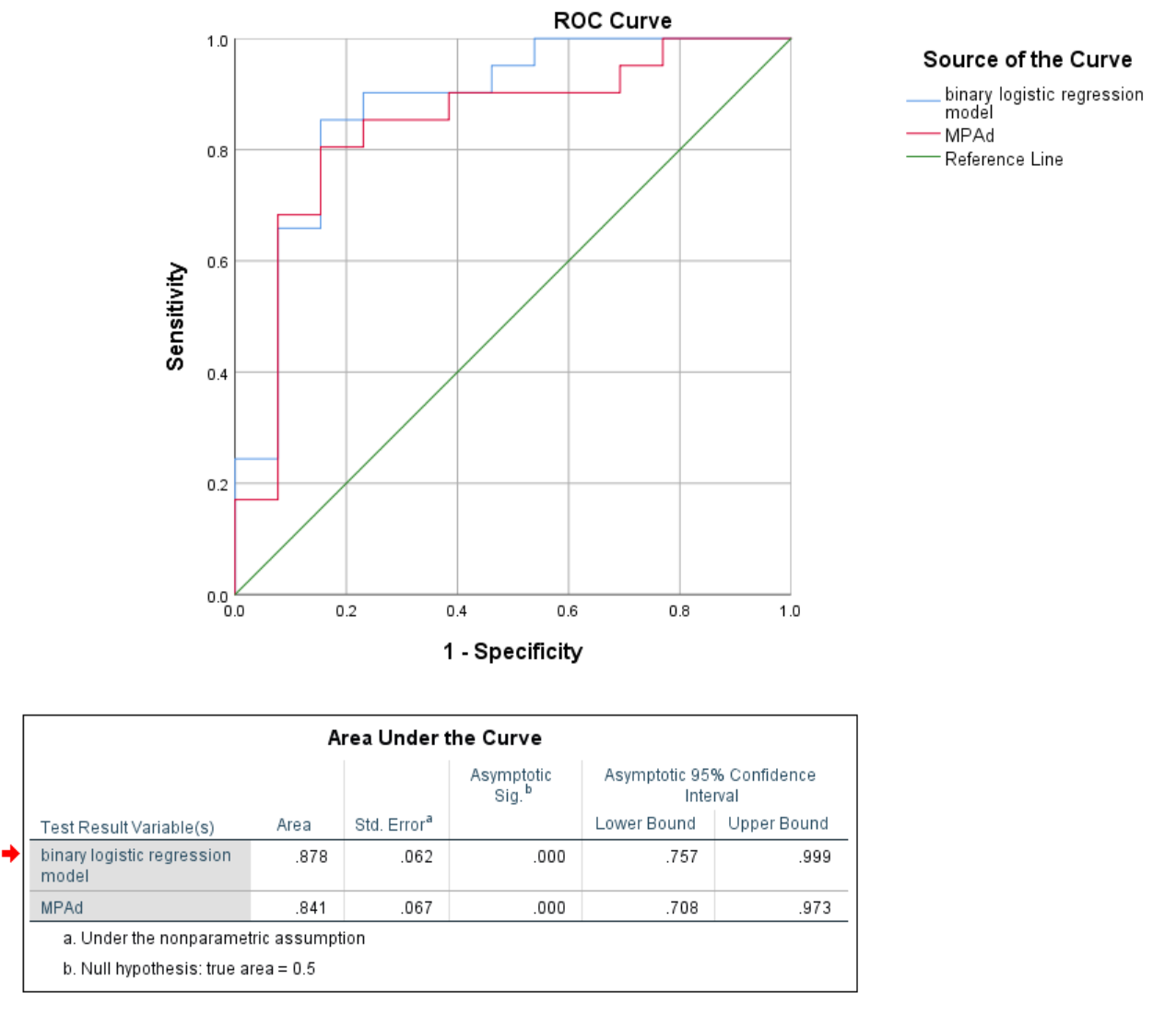


A. Area of ROC of binary logistic regression model and MPAd respectively is 0.878±0.062 and 0.841±0.067.


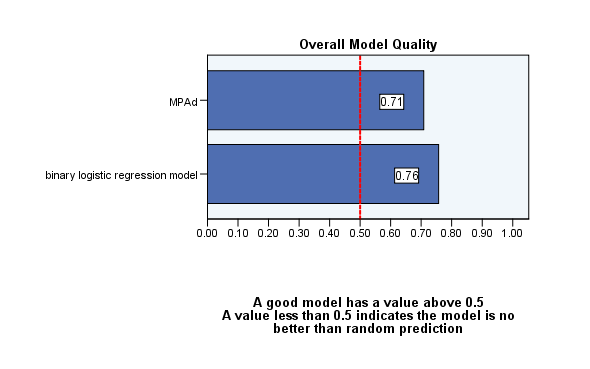


B. Over all model quality of ROC of the binary logistic regression model and main pulmonary arterial diameter (MPAd) respectively is 0.76 and 0.71.
